# Developing an Action Model for Integration of Health System Response to HIV/AIDS and Noncommunicable Diseases (NCDs) in Developing Countries

**DOI:** 10.5539/gjhs.v6n1p9

**Published:** 2013-10-08

**Authors:** Tilahun Nigatu Haregu, Geoffrey Setswe, Julian Elliott, Brian Oldenburg

**Affiliations:** 1 Department of Epidemiology and Preventive Medicine, Monash University, Australia; 2 School of Health Sciences, Monash University, South Africa; 3 Infectious Disease Unit, Alfred Hospital, Australia

**Keywords:** HIV/AIDS, Noncommunicable diseases, integration, model, health system response

## Abstract

**Introduction:**

Although there are several models of integrated architecture, we still lack models and theories about the integration process of health system responses to HIV/AIDS and NCDs.

**Objective:**

The overall purpose of this study is to design an action model, a systematic approach, for the integration of health system responses to HIV/AIDS and NCDs in developing countries.

**Methods:**

An iterative and progressive approach of model development using inductive qualitative evidence synthesis techniques was applied. As evidence about integration is spread across different fields, synthesis of evidence from a broad range of disciplines was conducted.

**Results:**

An action model of integration having 5 underlying principles, 4 action fields, and a 9-step action cycle is developed. The INTEGRATE model is an acronym of the 9 steps of the integration process: 1) Interrelate the magnitude and distribution of the problems, 2) Navigate the linkage between the problems, 3) Testify individual level co-occurrence of the problems, 4) Examine the similarities and understand the differences between the response functions, 5) Glance over the health system’s environment for integration, 6) Repackage and share evidence in a useable form, 7) Ascertain the plan for integration, 8) Translate the plan in to action, 9) Evaluate and Monitor the integration.

**Conclusion:**

Our model provides a basis for integration of health system responses to HIV/AIDS and NCDs in the context of developing countries. We propose that future empirical work is needed to refine the validity and applicability of the model.

## 1. Introduction

Integration is a dynamic and multidimensional concept. It may mean different things to different people. For some, integration is a polarized concept and for others it is a continuum. In Health systems, the term has been widely used to describe the bringing together of different components of the healthcare system. Integration is ‘a framework – a lens that can be systematically applied to better link units…it is a means to tackle fragmentation, duplication and gaps’ (Armitage, Suter, Oelke, & Adair, 2009). Though it could apply to any function/process in the health system, integration is commonly applied to service delivery to ensure *continuity* of services and thereby to effectively address patient needs (D.L. Kodner & Spreeuwenberg, 2002). The working definition of “integrated care” by the World Health Organization (2008) is “The management and delivery of health services so that clients receive a continuum of preventive and curative services, according to their needs over time and across different levels of the health system” This definition implies that integration is a function of both the management and the delivery of health services, and its purpose is to ensure continuity of services that is essential to address client needs.

**Figure 1:**
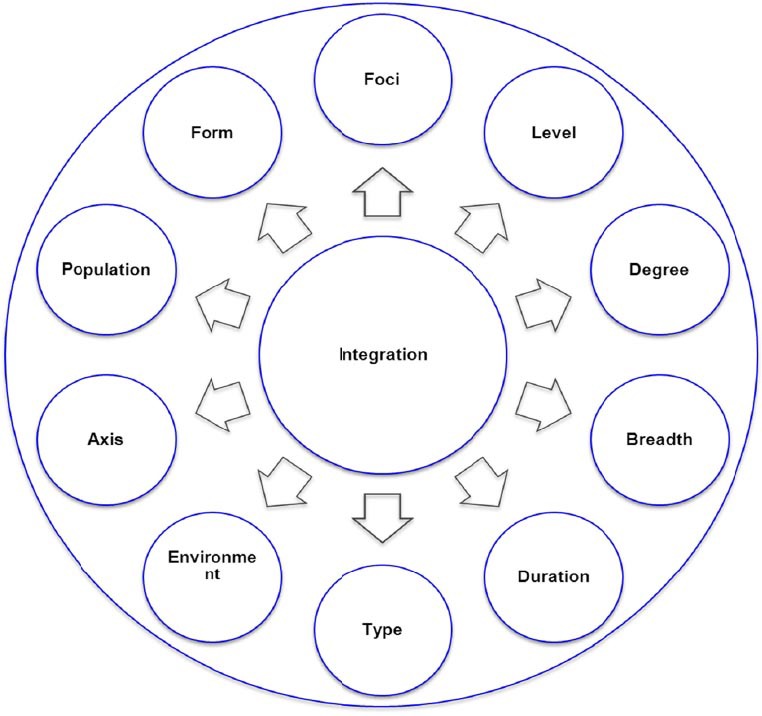
The ‘integration compact’

Integration is a means to an end rather than an end by itself (D.L. Kodner, 2009). Based on its different dimensions considered (‘integration compact’- See figure 1), there are different classifications of integration in a health system. According to the axis of the health system, integration could be *horizontal or vertical*. Depending on the level of health system where it occurs, integration could be *strategic* (upstream/Macro level), *managerial* (Midstream/Meso level) or *Operational* (Downstream/Micro level). Integration could also be partial or full; specific or generalized; strong or weak; localized or universal. Integration may also be classified based on the foci of integration, the units that are going to be integrated. Besides, integration can be internal (e.g. intra-program), external (inter-program), across stakeholders, across levels and with sector-wide responses.

Integration can be approached from a perspective of clinical model, business model, workforce model or community model (Karakusevic, 2010). Most of the currently existing integration models focus on the architecture of *output* of integration, the configuration of an integrated whole, and how it works (Lloyd & Wait, 2013). Some model address principles of successful health system integration (Armitage et al., 2009). Other studies addressing issues related to strategies of integration indicate how two services can be integrated (Dudley & Garner, 2011). There are many forms of health system integration models: System level (Lukas et al., 2002; Markoff, Finkelstein, Kammerer, Kreiner, & Prost, 2005; Miller, 2000), service level (Batterham et al., 2002; Byrnes, 1998; King & Meyer, 2006; O'Connell, Kristjanson, & Orb, 2000; Weiss, 1998; Wulsin, Sollner, & Pincus, 2006) and progressive models (Boon, Verhoef, O'Hara, & Findlay, 2004; Gillies, Shortell, Anderson, Mitchell, & Morgan, 1993; Leatt, Pink, & Guerriere, 2000; Leutz, 1999). Most of these models are service level models that focus on integration outputs rather than integration processes.

Health Systems in developing countries, especially in Sub-Saharan Africa, have been fighting the HIV/AIDS epidemic during the past three decades. Subsequently, several policies, programs, and systems have been developed to mitigate the impacts of this epidemic. As a result, the HIV/AIDS epidemic in those settings is stabilizing. However, the epidemic of Noncommunicable diseases (NCDs) is rapidly increasing in these countries. As HIV/AIDS and NCDs share many characteristics, an integrated approach could be a more effective and efficient alternative to curb the tide of the two epidemics. Given the political recognition of the need for integrated approach and the resource constraints in developing countries, there is an urgent need for evidence-based models for integrating the responses to HIV/AIDS and NCDs.

However, to the knowledge of the authors, there is no systematic and systemic model that could guide the *process* of integrating two or more health system response functions like that of HIV/AIDS and NCDs. Therefore, this study was designed to develop an action model, a step-by-step approach, for the integration of health system responses to HIV/AIDS and NCDs in the context of developing countries. The new model is expected to be useful for assessment, strategy development, healthcare management and in refinement of existing strategies related to integration.

## 2. Model Development Methods

Multiple stages and steps were involved in this study. We began this study by carrying out a *scoping review* of the literature to explore the rationale for integrating the responses to HIV/AIDS and NCDs in developing countries (T. Nigatu, 2012). This initial review revealed that analysis of the *connections* between HIV/AIDS and NCDs is essential to establish the need for an integrated response. Accordingly, we conducted analyses and synthesis of the connections between HIV/AIDS and NCDs at three levels: Population, Disease and Individual level. After the analysis of the connections, it became evident that an additional dimension, analysis of the relationships between the responses to the HIV/AIDS and NCDs, is essential to identify the eligible functions for integration. Accordingly, we conducted a qualitative analysis of the similarities and differences between responses to HIV/AIDS and NCDs case study approaches. Further exploration of evidence from integration of HIV/AIDS and reproductive health services in developing countries yielded an additional factor important for our model development process. This was the role of organizational/system *environment*. In this study, we described the major ‘environmental’ factors that should be considered in the integration process.

Following the generation of evidence for integration, the next logical step is communication. In order for evidence to better inform decisions, it needs to be *repackaged* in to a more simple and understandable form. By adopting concepts from knowledge translation models, we included this important step in to our model. Finally, we used the PIE (Plan, Implement and Evaluate) model of integration management for the rest of the steps. We detailed the application of all the steps in using the HIV/AIDS and NCD integration as a working example.

In overall, this model development process was *iterative* and *progressive*. We used descriptive, integrative and configurative evidence synthesis techniques at different levels. We considered the model to be *cyclical* rather than *linear*. The primary information sources during the design of the model were the sub-studies conducted as part of a larger research project and additional trans-disciplinary literature review.

## 3. The INTEGRATE Model for Integration

Integration consists of a series of steps that *integration managers* should implement to arrive at an effective integration of functions. If *integration managers* use a systematic process of integration, there will be a higher chance of successful integration as compared to the use of fragmented and unsystematic processes of integration.

This article describes a model for integration process of health system response functions of HIV/AIDS and Noncommunicable diseases using the acronym INTEGRATE. Each letter of the acronym INTEGRATE stands for a step in the action cycle that needs to be carried out before proceeding to the next step. The nine steps in the INTEGRATE Model are shown in the figure below.

**Figure 2:**
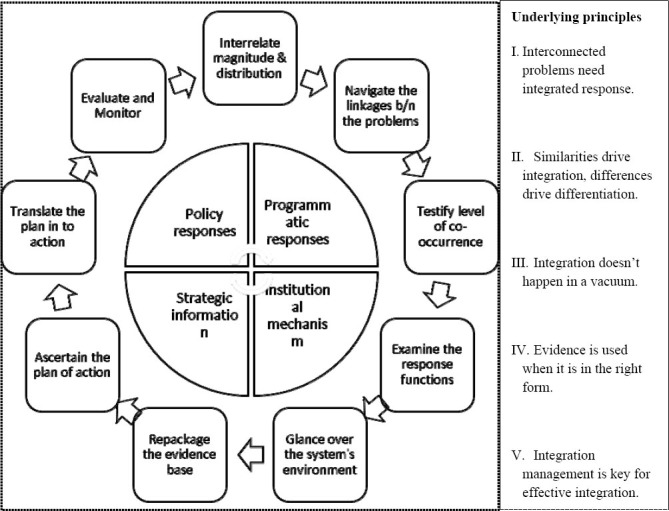
INTEGRATE: Integration action model with 5 principles, 4 action fields and a 9-step action cycle

As described in the above figure, the resulting action model has five underpinning principles, five key processes, four action fields and a nine-step action cycle. These principles, key processes and steps are illustrated in the following sections.

### 3.1 Interconnected Problems Need Integrated Response

Key process I: Analyze connections between the problems

As it is the case for many other management functions, integration starts with understanding of HIV/AIDS and NCDs, more specifically the connections between them. Using the *syndemic* theory and ecological model, analysis of the connection between HIV/AIDS and NCDs can be conducted at three levels: Population, Problem and Individual levels.

Step 1: Interrelate the magnitude and distribution of the problems

Interrelating the magnitude and distribution (by person, place and time) of HIV/AIDS and NCDs at *population* level is essential to identify epidemiological overlaps between the problems. Evidence from population level overlap informs overall policy approaches and priorities. It also informs the need for a more detailed analysis. The magnitude of HIV/AIDS and NCDs can be interrelated at point in time (in a cross-sectional manner), across time (in a time-series manner), across different geographic settings, and across different segments of a population. The interrelationships between *patterns and trends* of HIV/AIDS and NCDs across different dimensions could be explored using a cluster analysis, trend analysis and correlational analysis techniques.

In the analysis of epidemiological *overlap* between HIV/AIDS and Diabetes (considered as a prototype NCD) in developing countries, Diabetes was found to have higher prevalence and HIV was found to have higher disability-weight-adjusted prevalence. Diabetes had an increasing trend while HIV had a stabilizing trend. Countries with high diabetes prevalence tend to have lower HIV prevalence. Four clusters of countries were identified from the cluster analysis of the patterns in the prevalence of HIV/AIDS and Diabetes (Haregu, Elliott, Setswe, & Oldenburg, 2013).

Step 2: Navigate the linkage between the problems

At disease level, analysis of the *linkage* between HIV/AIDS and NCDs and plotting the linkage in to a framework is important for better understanding the interconnections between the diseases. This evidence is essential for a coordinated response to public health problems as well as in developing the content of intervention packages. The *linkage* between HIV/AIDS and NCDs has direct or indirect pathways and is based on both *risk* and *severity*.

In the development of a public health framework for the epidemiological linkage between HIV/AIDS and the common NCDs (Cardiovascular disease, cancer, Diabetes and chronic obstructive pulmonary disease), six major pathways of linkage were found. These were direct cause-effect relationship, common underlying/background factors, lifestyle factors, treatment effects, common complications, and other disease conditions (T. Nigatu, Setswe, Elliott, & Oldenburg, 2012). Apart from these, similarities in disease characteristics are also important.

Step 3: Testify individual level co-occurrence of the problems

The third level in the analysis of the connections between HIV/AIDS and NCDs is their level of co-occurrence - (Comorbidity and Multimorbidity). This is analysis of individual level co-occurrence of the health problems. This evidence is important in planning and resource allocations. As most health systems in developing countries can’t afford to assign more than a single health care provider (team) to manage a patient with comorbidity and patients in those settings have the right to receive all the healthcare services they need from a single service delivery point, analysis of magnitude of co-occurrence of HIV/AIDS and NCDs is important to design effective strategies that can address the needs of multi-morbid patients.

Perspectives, constructs, and methods in the measurement of comorbidity and multimorbidity are detailed elsewhere (Haregu, Oldenburg, Setswe, & Elliott, 2012). In our analysis of the magnitude of comorbidities of common NCDs among HIV positive people, we have documented evidence of increased risks of cancer and cardiovascular diseases and the absence of evidence of increased risk of diabetes (Haregu, Oldenburg, Setswe, Elliott, & Nanayakkara, 2012; T. Nigatu, Oldenburg, Elliott, Setswe, & Woldegiorgis, 2013). Anti-retroviral treatment (ART) is associated with decreased magnitude of NCD comorbidity among HIV+ people (Islam, Wu, Jansson, & Wilson, 2012).

### 3.2 Similarities Drive Integration, Differences Drive Differentiation

Key process II: Examine the similarities and differences between responses

Step 4: Examine the similarities and understand the differences between response functions

After establishing the need for integrated response through analysis of connections between HIV/AIDS and NCDs, the next step is to identify eligible units for integration by examining similarities and differences between response functions. This step has four sub-steps.

#### 3.2.1 Identify the Response Functions (i.e. Action Fields)

How health systems are responding to HIV/AIDS and NCDs? What constitutes a Health system response to HIV/AIDS and NCDs? What are the major processes/functions in a health system response? There are different classifications of the functions of a health system including the WHO Health system framework (World Health Organization). Qualitative analysis of the similarities and differences between responses to HIV/AIDS and NCDs revealed four inter-related categories of functions (Haregu, Oldenburg, Elliott, & Setswe, 2013).

These were *policy* related functions, *program* related functions, institutional arrangement/*coordination/* related functions, and *strategic information* related functions. Analysis of parallels and differences between the national responses to HIV/AIDS and NCDs yielded a fifth additional category which was labelled as *management* related functions. The functions under these major categories are shown in the table below.

**Table 1:** List of major functions in a health system response

Categories	Functions	Description of the functions
Policy	Leadership	High level political commitment
	Policy advising	Providing inputs for policy making
	Policy making	Formulation of policies
	Governance	Overseeing policy implementation processes
Program	Prevention	Measures taken to prevent disease
	Treatment	Services provided to control/treat disease
	Care & support	Services provided to improve quality of life
	Health System Strengthening	Interventions that improve system capacity
Management	Planning	Strategic and annual planning
	Implementation	Overseeing implementation of national programs
	Resource Mobilization	Securing resources needed for programs
	Multisectoral. Coordination	Coordination of multiple actors/sectors
Strategic information	Patient Monitoring	Monitoring the progress of patients
	Disease Monitoring	Monitoring of disease/epidemic patterns
	Program Monitoring &Evaluation	Monitoring and Evaluation of national programs
	Dissemination of information	Communication of findings of M&E

The scope and details of the above functions would vary across the three levels of the health system: Strategic (Macro), Managerial (Meso) and Operational (Micro). When one goes upwards in a health system, programmatic/technical/functions get more *specialized* and administrative functions get more *converged*. In the process of integration, these functions/processes are integrated to arrive at an integrated whole.

#### 3.2.2 Set Parameters to Compare Response Functions

Once the functions relevant for integration management are identified, the next step is to assess the relational similarities (and differences) between these functions. The basic question then will be ‘what parameters should be used to assess the similarities between response functions?’ These are the comparators, instruments for comparison. Below is a list of possible comparators. Although all these comparators may not apply to each and every function, the list is believed to include most of the relevant comparators.

**Table 2:** List of possible parameters for assessing the similarities between functions

Parameters (similarity in what?)	Descriptions
Operational characteristics	Nature and technical complexity
Timing of the functions	Time and frequency (when and how often)
Actors/performers	The skills/expertise/speciality required
Methods/tools	Models and approaches used
Targets/users	The characteristics of the customers/users
Results/outputs	The attributes of the end products
Input requirements	Financial and non-financial requirements
Levels in the system	Levels of health system where the functions happen
Lines of accountability	Command and communication chains
Monitoring Modalities	Monitoring requirements (formats, schedules etc)
Priorities and interests	Accorded priorities and vested interests

#### 3.2.3 Rate the Degree of Similarity/Difference between the Response Functions

In a health system response to a HIV/AIDS and NCDs, it is possible for some functions to be more or less integrated than others. Though any set of functions can be assessed for degree of similarity and thus be eligible for integration management, the practice is common for parallel functions (e.g. treatment of HIV and Treatment of NCD, rather than treatment of HIV and Prevention of NCD). The degree of similarity between response functions can be rated using either a dichotomous or a likert-type scale. Below is a five point likert-type scale for rating two functions using the parameters identified in the above steps. The scores can be summed up to get a single summary score.

Who should rate the similarities? We propose two approaches: 1) The *deliberative* approach where a panel of experts, preferably from the concerned functions, deliberate on the similarities and provide an agreed up on score; 2) The *investigative* approach where an investigator assess the similarity between the functions and give a score based on the findings.

#### 3.2.4 Determine the Strategic Importance of the Similarities/Differences

In addition to the degree of similarity, the importance (strategic, managerial, economical and operational) of the similarities and differences between functions is essential for integration. Finally, the examination of similarities/differences should come up with short list of functions that can undergo the integration process.

**Table 3:** Rating similarity between two functions using the assessment parameters

SN	Parameters for similarity (Similar in what?)	Degree of Similarity (How much similar?)				
		Very Low (1)	Low (2)	Medium (3)	High (4)	Very High (5)
1	Technical characteristics
2	Priorities/premises
3	Actors/performers
4	Methods/tools
5	Targets/users
6	Results/outputs
7	Settings/contexts
8	Stages of Maturity
9	Lines of accountability
10	Monitoring Modalities
	Total score

### 3.3 Integration Doesn’t Happen in a Vacuum!

Key process III: Scan the health system environment for integration

Step 5: Glance over the Health system’s environment for integration

As integration happens in the real environment, the other major factor that is important for integration is the health system environment, the set of forces surrounding the health system that have the potential to affect the way it operates and its access to scarce resources. These include *internal* environment – internal to the units to be integrated (e.g. healthcare workers, managers, healthcare settings), the *task* environment (e.g. patients/clients, other actors, partners, donors, pressure groups), and the *external* environment (Political, Economic, Socio-cultural and Technological factors).

Among the prominent system factors in the context of developing countries is motivation among policy makers, health managers and providers for integration. *Acceptability* of the level of integration by target users is also important. Other important elements are capacity for integration, external influences of different stakeholders on integration and the impacts of integration on important stakeholders.

**Figure 3:**
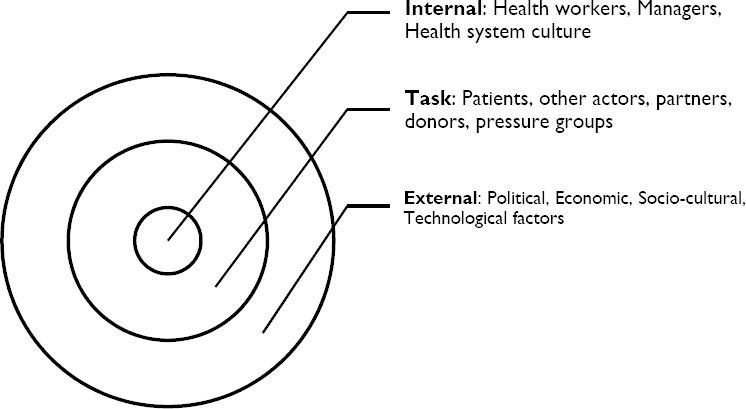
Elements of Health system environment

### 3.4 Evidence is Used Only When it is in the Right Form

Key process IV: Repackage evidence for integration

Step 6: Repackage and share evidence in a usable form

In Health systems integration, the focus has primarily been on structures. Though equally important systems level integration of functions has received little attention (Burns et al., 2001; Fawcett & Cooper, 2001). For system level integration of health system responses, different integration strategies need to be employed at multiple levels (D. L. Kodner, 2002). Similarly, multiple processes have to be used to ensure successful integration. Integration strategies at *system* level and *service* level are different (Dodds et al., 2004). *System level* strategies relate to administrative linkages between partners, shared philosophy statement, and role enhancement of certain provider groups (Wilson, Rogowski, & Popplewell, 2003). Accordingly, evidence for system level integration should be communicated to different audience at multiple levels. As different audience have different interests, the evidence needs to be repackaged before it gets disseminated.

Evidence repackaging is the presentation of evidence in a more understandable, readable, acceptable and usable form. The contents of the evidence obtained from the analyses in the above steps would indicate the need for integration, eligible functions for integration, various factors that would affect integration, *desired* level of integration, *existing* level of integration and the *gaps* between the desired and existing levels of integration. Purpose-driven packaging of evidence can facilitate effectiveness of communication.

The content of evidence relevant to integration can be repackaged based on the models of integration. The model for system level integration depends on the required level of integration, and resources available for conducting the integration process and executing the integrated functions. The following are examples of models of integrating functions: a *causal* *model* of organizational performance and change (Burke & Litwin, 1992), a *3-dimensional* *model* developed based on key dimensions of an integrated health system (Conrad & Shortell, 1996), a *linear model* developed to measure outcomes (Lukas, et al., 2002), and the Relational Systems *Change Model* developed based on psychological development (Markoff, et al., 2005).

Moreover, evidence about integration of functions could also be repackaged based on desired types of integration: Virtual (networked) integration, vertical (hierarchical) integration, horizontal (same level) integration, functional integration, clinical integration, and physician integration. In general, repackaging of integration related evidence should consider the audience, the content, the purpose and the method of communication.

### 3.5 Integration Management is Key to Effective Integration

Key process V: Manage integration

Step 7: Ascertain the plan of integration

Levels of integration can be considered as parts of a continuum. There are several models of levels of integration that are developed based on health service integration (Heath, Wise Romero, & Reynolds, 2013; Suter et al., 2007). It is possible to assume that there is always some level of integration between two functions. Identifying the existing level of integration between two functions is the first task at this step. The next task is determining the desired level of integration between the functions. This will be the aim of the integration process.

Integration managers should develop a detailed Policy/Strategy/*Plan of integration* that describes the components of responses are going to be integrated, the existing and the desired level of integration, the strategies to be used, the resource requirements, and the benefits and the risks of the integration. This will serve as a roadmap for the overall integration process. Assessment of local and global *experiences* and *best practices* relevant to the integration is essential. Communication and coordination (e.g. through consultative meetings with relevant stakeholders) is very crucial at this stage. Measurable objectives should be developed and agreed up on. Contextual factors, like personal preferences and urgency of the problem and interests of the various stakeholders involved may affect the planning and implementation integration. The required level of system integration will also depend on the type of interdependence (pooled, sequential, or reciprocal) in the structure of the systems (Peters, 1998). The resulting integration plan needs to be aligned with existing policies and strategies.

**Evidence about HIV-NCD integration in the literature**

1. Cervical Cancer screening in to HIV services (Sneden, Huchko, Cohen, & Yamey)
2. Gestational Diabetes screening in to HIV treatment/PMTCT (Gonzalez-Tome et al., 2008)
3. HIV/AIDS, Diabetes, and Hypertension services in to a chronic disease clinic (Janssens et al., 2007)
4. Leveraging HIV programs to support diabetes services (Rabkin et al., 2012)
5. Integrating smoking cessation in to HIV care -(Drach et al.)
6. Integrating HIV/AIDS and Alcohol (Bryant, Nelson, Braithwaite, & Raoch, 2010)

The selection of approach of integration depends of local contexts. For instance, policy integration of HIV/AIDS and NCDs can be realized in terms of one policy governing both HIV/AIDS and NCDs or a special policy guiding the integration of HIV/AIDS and NCD responses. Policy statements that support the integration of HIV/AIDS and NCD responses could also be included in both HIV/AIDS and NCD policy documents. Such policies/strategies could guide integration at program, management and information levels.

**Table 4:** Possible levels of integration of response functions

S.N.	Levels	Description of levels
1	Communication	Exchange of information, keep up to date
2	Consultation	Informing actions and consulting for actions
3	Coherence	Ensuring that there are no contradictions in actions
4	Consensus	Recognizing interdependence and ensuring harmonization
5	Coordination	Helping each other but not changing the basic way of doing business
6	Cooperation	Helping each other in specific ways
7	Collaboration	Working together/jointly on mutual interests
8	Co-location	Functions co-located to be under the same space/facility
9	Coalition	Performing functions with the same team with role difference
10	Combination	Transforming functions in to a single merged practice

Step 8: Translate the plan to action

The action fields for Health system response are identified as policy response, programmatic response, institutional mechanism, and strategic information. The integration strategies to be used can vary based on both the action field and the desired level of integration. The common system level strategies in the implementation of health system integration include patient engagement and participation, well-developed performance management system, well-functioning information system, cohesive organizational culture, relationship development, right sizing and controlled growth, and sound financial management. The level, type and combination of strategies required are highly contextual and have to be geared towards better outcomes. System level integration may begin at any level. But it must extend to all other levels. Integration between two functions could be at the development of a plan, implementation of activities and at Monitoring and Evaluation of a program.

Successful implementation of integration requires time, sufficient resources and a defined, multi-model intervention. A formal Strategic plan with measurable objectives, time lines, responsibilities, and outcomes is essential. Structural changes and resource sharing along with sufficient attention to relationships are required at all levels of a system. Flexibility of leadership is essential to adapt to the changes. Development and execution of strategies is progressive. Never expect to “finish” the integration – Integration is an on-going process (Contandriopoulos, Denis, Touati, & Rodriguez, 2003; Vanderbent, 2005).

Barriers to effective integration are categorized in to *specialization* barriers and *political* barriers. In contrast, mechanisms that facilitate the implementation of integration include standardization (work, output, skill and norms), direct supervision, coordinated planning, and mutual adjustment. The suitability of the mechanism of integration depends on task *complexity* and task *interdependence* (Barki & Pinsonneault, 2005).

Step 9: Evaluate and Monitor the integration

Evidence related to evaluation of integration is related to the following constructs: Configuration, Effectiveness, Efficiency, Synergy and impact. *Configurations* are forms of alignment between strategy, structure, processes and environment. Measurement of integration (the level/intensity of integration of integrated system – the actual degree of integration between functions) often refers to configuration. Integrated policies, integrated programs, integrated management, integrated organizational structures and integrated monitoring and evaluation systems are the outputs of strategic level integration. The changes in configurations are expected to reduce *complexity* but may increase *connections*. Configurations in most models of degree of integration consider a continuum extending from full segregation through a number of intermediate forms to full integration (Ahgren & Axelsson, 2005).

*Effectiveness* of integration refers to the achievement of the intended goals of the integration. There is a difference between the achievement of the goals of the health system (impact) and the achievement of the goals of integration (effectiveness). The change in *performance* of a system that could be attributed to the integration process is the effect of the integration. Accordingly, integration effectiveness can be measured at community, integration/network and organization levels (Provan & Milward, 2001). The stakeholder groups and effectiveness criteria are different among these levels.

*Synergy* among the integrated functions is another important *proximal* outcome of integration. Synergy in the context of integrated functions means the “combined effect” of multiple functions as compared to the sum of the effect of the independent functions. Synergy among functions is influenced by resources, partnership characteristics, relationship among partners and external environment (Lasker, Weiss, & Miller, 2001).

*Efficiency* of integration is often related to the financial performance in the process of achievement of the intended outcomes of the health system. However, efficiency of integration should be viewed from a broader perspective. In Health system context, efficiency refers to how well healthcare resources are used to obtain health improvements. This comprises of two components: *Technical* efficiency (whether functions are performed with the least amount of inputs) and *allocative* efficiency (whether a set of technically efficient functions is chosen to yield the greatest possible outcome) (Peacock, Chan, Mangolini, & Johansen, 2001).

**Figure 4:**
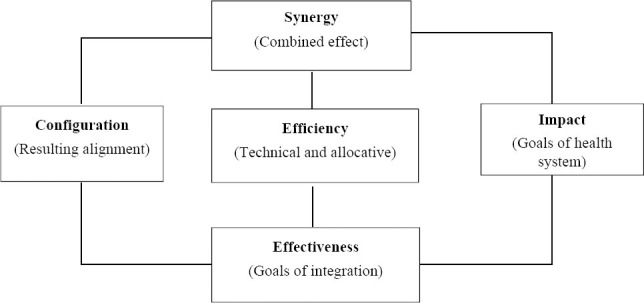
A framework for Evaluation of integration

The widely used measurement tool for integration is the *Balanced Score Card* (BSC). BSC is appropriate to evaluate both the implementation and impact of integration (Kaplan & Norton, 1993). The second measurement tool is the *clinical microsystem assessment* tool (Peacock et al., 2001). This tool is primarily designed for the clinical system. The third tool is the *scale of functional integration* which is validated for several forms of integration (Nelson et al., 2002).

## 4. Conclusion

Developing countries are facing a double-burden of HIV/AIDS and Noncommunicable diseases. As the intersection between HIV/AIDS and NCDs is significantly high, an integrated response can maximize synergy and improve efficiency of the responses. The need for integrated response is therefore clear.

Integration is an iterative, dynamic and complex process. Using synthesis of evidence from various fields, we have developed an action model that could guide the integration of health system responses to HIV/AIDS and NCDs in developing countries. The model has 5 underpinning principles, 5 key processes and 9 steps. It could serve as a step-wise framework for systematic integration of health system responses to HIV/AIDS and NCDs in the context of developing countries. Although this model is prepared as an action cycle, users may use the steps out of sequence, as appropriate, depending on their context. The model can be a good basis for the development tools that could be used to operationalize the different steps stated in this model.

This model is developed based on synthesis of best available evidence about integration of responses to HIV/AIDS and NCDs. The principles and the steps developed in this action model could be adapted to other disease conditions in similar contexts. This action model is conceptually enriched through a review of literature from different disciplines. However, it hasn’t yet been empirically tested. Besides, the steps in this model are stated as general guiding statement and are not operationalized in to specific tasks and activities.

Therefore, we propose that future empirical work is needed to refine the validity and applicability the model. The model should also be translated in to tools that facilitate its application to meet the intended purpose. Further research work is also needed to refine this model.
